# Hamlyn CRM: a compact master manipulator for surgical robot remote control

**DOI:** 10.1007/s11548-019-02112-y

**Published:** 2020-01-19

**Authors:** Dandan Zhang, Jindong Liu, Lin Zhang, Guang-Zhong Yang

**Affiliations:** 1 The Hamlyn Centre for Robotic Surgery, Imperial College London, London, SW7 2AZ UK; 2 Present Address: Precision Robotics, 80 Wood Lane, London, W12 0BZ UK; 3 The Institute of Medical Robotics, Shanghai Jiao Tong University, Shanghai, China

**Keywords:** Surgical robot, Master manipulator, Teleoperation

## Abstract

**Purpose:**

Compact master manipulators have inherent advantages, since they can have practical deployment within the general surgical environments easily and bring benefits to surgical training. To assess the advantages of compact master manipulators for surgical skills training and the performance of general robot-assisted surgical tasks, Hamlyn Compact Robotic Master (Hamlyn CRM) is built up and evaluated in this paper.

**Methods:**

A compact structure for the master manipulator is proposed. A novel sensing system is designed while stable real-time motion tracking can be realized by fusing the information from multiple sensors. User studies were conducted based on a ring transfer task and a needle passing task to explore a suitable mapping strategy for the compact master manipulator to control a surgical robot remotely. The overall usability of the Hamlyn CRM is verified based on the da Vinci Research Kit (dVRK). The master manipulators of the dVRK control console are used as the reference

**Results:**

Motion tracking experiments verified that the proposed system can track the operators’ hand motion precisely. As for the master–slave mapping strategy, user studies proved that the combination of the position relative mapping mode and the orientation absolute mapping mode is suitable for Robot-Assisted Minimally Invasive Surgery (RAMIS), while key parameters for mapping are selected.

**Conclusion:**

Results indicated that the Hamlyn CRM can serve as a compact master manipulator for surgical training and has potential applications for RAMIS.

## Introduction

A typical robotic surgery system consists of three major components: a master control console with interactive manipulators, a slave surgical robot with articulated instruments and a feedback system with vision and other sensing modalities. There is a tendency that surgical robotic platforms are developing toward smarter and smaller systems in recent years [1]. Compact master manipulators can occupy less space and are more affordable, which are worth developing.

**Fig. 1:**
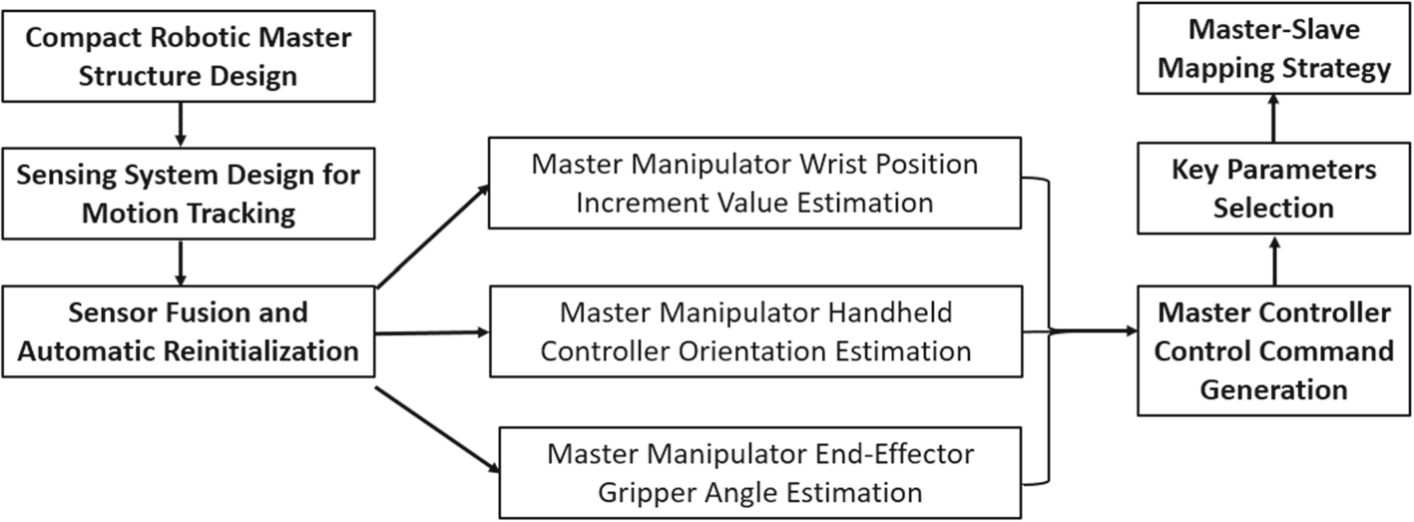
The overview of the design workflow of a new compact master manipulator

Master–slave control is a form of teleoperation where the slave robot is controlled by mimicking the surgeons’ movements. To ensure safety and accuracy for surgical robot remote control, precise motion tracking for the master manipulator is significant, which can affect the quality of robotic surgery. With accurate motion tracking techniques, the slave robot can exactly relay the commands from the operator to fulfill the surgical tasks with high efficiency, which serves as a fundamental part in a teleoperation system. For surgical robot remote control, most of the existing master manipulators utilize mechanical structures to realize motion tracking. For example, the control console of the da Vinci robot employs a serial-link manipulator for teleoperation [2]. The mechanical linkage with the remote center parallel mechanism can be used to transmit commands from an operator to a slave robot with high accuracy. Potentiometers or encoders are used to monitor the joint angles and obtain the end-effectors’ position and orientation based on the transform relationship. It is stable and reliable, but not appropriate for master manipulator compact design. To this end, other suitable motion tracking techniques should be explored.

Existing motion capture techniques which do not impose too much restriction on the operations are based on visual, inertial, optical, electromagnetic, acoustic systems or other hybrid techniques. Advantages and disadvantages of different motion tracking systems have been summarized in [3]. Considering that different modalities have different pros and cons, hybrid methods can be developed to combine the advantages of different methods and compensate their drawbacks at the same time. Therefore, sensor fusion is used to enhance the tracking accuracy of the compact master manipulator in this paper.

Master–slave mapping is significant to be considered for teleoperation [4]. For the human-in-the-loop control, visual feedback is important for control efficiency, which has a significant influence on hand–eye coordination. Moreover, the measured motion of the master manipulators is scaled-down and replicated by the slave robot during teleoperation [5]. An appropriate motion scaling ratio should be determined for the master–slave strategy. The kinematic correlation between the master controller and the slave robot is also an important aspect to be considered. For master–slave mapping, comparison of the position mapping mode (P Mode) and the velocity mapping mode (V Mode) for efficient robotic endoscopy has been conducted in [6]. However, it focuses on the 3D position mapping, while the 3D orientation mapping for articulated instruments was not discussed. Hand–eye coordination, as well as the selection of a reasonable motion scaling ratio, was not analyzed to form a complete mapping relationship for surgical robot remote control. Therefore, a suitable mapping strategy is explored in this paper, taking all the aforementioned factors into consideration.

Compared to the conventional mechanical-linkage-based robotic master, the compact master manipulator has a special sensing system. In addition to structure design, motion tracking and master–slave mapping are significant for the design of a new master manipulator. Therefore, the key parameters selection and mapping strategy for teleoperation should be explored based on the proposed master manipulator. The overall workflow for the design of a new compact master manipulator is shown in Fig. 1.

In this paper, we introduce the Hamlyn CRM, which is a compact master manipulator for surgical robot remote control. Firstly, the details of the structure design are introduced in “Hardware design” section. Subsequently, the sensing system for motion tracking is illustrated and the sensor fusion method with an automatic reinitialization mechanism is described in “Motion tracking” section. In “Mapping strategy” section, user studies were conducted on the da Vinci Research Kit (dVRK) by controlling the slave robot via the Hamlyn CRM. The user studies were targeted for the decision support of the key parameters determination, the master–slave mapping strategy selection and the usability verification. Finally, discussions were carried out in “Discussion” section, while conclusions were drawn in “Conclusion” section.

**Fig. 2:**
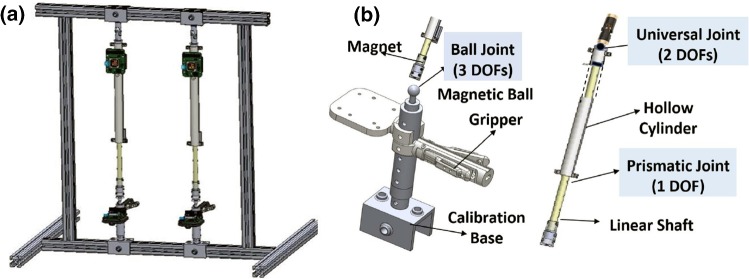
CAD model of the Hamlyn CRM. **a** Overview of the overall structure. **b** Structure details illustration

## Hardware design

### Structure design

The translation and orientation components can be independently controlled, since a position and orientation decoupling design is employed for the Hamlyn CRM. The overall CAD model of the Hamlyn CRM and the detailed structure illustration are shown in Fig. 2. The prototype is composed of a handheld end-effector controller and a two-stage linear linkage, which are used to sense the hand’s orientation and position, respectively. The two-stage linear linkage consists of a universal joint and a prismatic joint. In order to reach the target of compact master manipulator design, the current prototype of Hamlyn CRM is a passive robotic arm without motors. Since the overall moving weight of the master manipulator is minimized to 300 g, which is lightweight enough, the gravity compensation is not necessary.

For orientation control, most of the master manipulators employ a gimbal mechanism, such as the Phantom Omni and the dVRK Master Tool Manipulators (MTMs). Gimbal mechanism consists of three joints with axes intersected at one point, which can provide enough flexibility and enable the operators to have a sense of natural control. To have a compact design, ball joints are applied for serial rotation as a 3-DoFs joint. It is realized by a magnetic ball connection between the end effector and the linear linkage with a magnet at the end. A one-DoF gripper, which is mounted on the handle of the handheld end-effector controller, is used to control the jaw of the slave robot.

For the position control, a universal joint is utilized as a 2-DoFs joint, which is more compact than the traditional method of combining two single rotational joints. Linear motion is generated by a linear shaft and a hollow cylinder. The universal joint and the prismatic joint are employed to provide position control in the spherical coordinate.

### Kinematics for the prototype

**Fig. 3:**
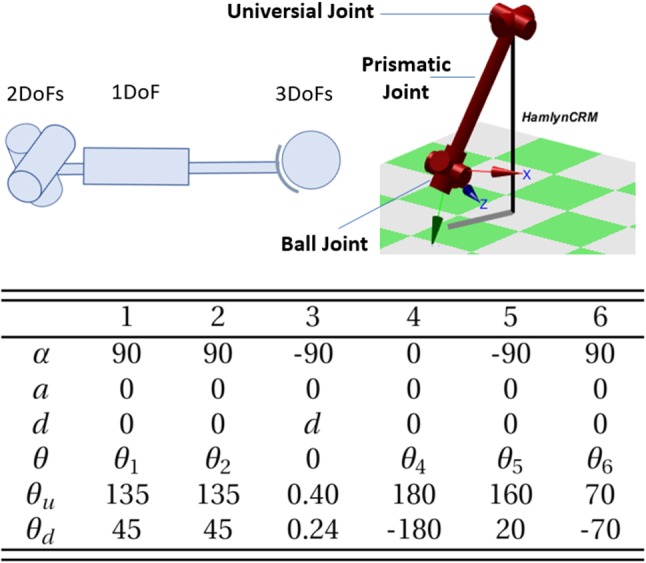
The sketch of the kinematic structure of the Hamlyn CRM; the DH Table and the Joint Limits for the Hamlyn CRM

The DH table and joint limits of the proposed master manipulator (the Hamlyn CRM) are shown in Fig. 3, where $\theta {_i}(i=1,2,4,5,6)$ and *d* represent the joint variable, ${\theta }_u$ and ${\theta }_d$ represent the upper and lower joint limit, respectively. The unit for the angle of the revolute joint is degree, while the unit for the link offset and the joint limits for the prismatic joint is meter.

**Fig. 4:**
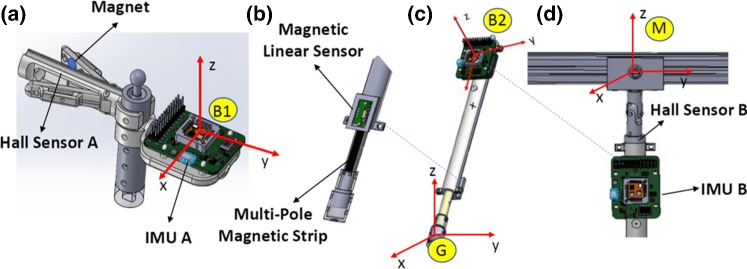
Overview of the sensing system for the Hamlyn CRM. Sensing system for **a** end effector; **b** prismatic joint with linear motion sensor; **c** IMU sensor for the two-stage linkage orientation sensing; **d** grounding method of the universal joint to base frame M

The sketch of the kinematic structure of the Hamlyn CRM, and the DH Table of the Hamlyn CRM are shown in Fig. 3. $$T_i^{i-1}$$ represents the transform matrix from the frame of the $${i-1}$$th joint to the frame of the *i*th joint. Based on the DH table, $$T_4^1$$ is the homogenous transform matrix from the base to the end effector, which can be obtained by $$T_4^1 = {T_4^3} {T_3^2} {T_2^1} $$. The final mathematical expression of $$T_4^1$$ can be found in (1), where the coordinate of the first joint is coincided with the frame of the base frame.

1 $$\begin{aligned} T_4^1=\begin{bmatrix} c(\theta _1)c(\theta _2) &{} s(\theta _1) &{} c(\theta _1)s(\theta _2) &{} c(\theta _1)s(\theta _2)d \\ s(\theta _1)c(\theta _2) &{} -\,c(\theta _1) &{} s(\theta _1)s(\theta _2) &{} s(\theta _1)s(\theta _2)d \\ s(\theta _2) &{} 0 &{} -\,c(\theta _2) &{} -\,c(\theta _2)d\\ 0 &{} 0 &{} 0 &{} 1 \end{bmatrix} \end{aligned}$$

Therefore, the position vector of the end effector ${\varvec{P}}_{\varvec{e}}$ = [$X_\mathrm{e}$, $Y_\mathrm{e}$, $Z_\mathrm{e}$] can be obtained as follows.

2 $$\begin{aligned} {\left\{ \begin{array}{ll} X_\mathrm{e} = \hbox {cos}(\theta _1)\hbox {sin}(\theta _2)d\\ Y_\mathrm{e} = \hbox {sin}(\theta _1)\hbox {sin}(\theta _2)d\\ Z_\mathrm{e} = -\,\hbox {cos}(\theta _2)d \end{array}\right. } \end{aligned}$$

## Motion tracking

### Pose estimation with multiple sensors

For portable applications based on inertial measurement, using additional sensors to help improve the measurement accuracy is common, while adding magnetometer as a supplement seems to impose the minimal constraints on the application. 9-axis IMUs combine a 3-axis magnetometer with inertial sensors can provide a complete measurement of orientation relative to the gravity direction. After fusing the sensor information generated by the accelerometer, gyroscope and magnetometer, the stable and accurate orientation estimation can be realized.

The sensing system for the Hamlyn CRM includes two 9-axis IMUs (MTi-3, Xsens), two Hall sensors (SS495A1) and one magnetic linear sensor (NSE5310) for each manipulator. The magnetic linear sensor is an incremental position sensor with on-chip encoding for direct digital output. A Hall element array on the chip is used to derive the incremental position of an external magnetic strip. With better than 0.5 micron resolution, the magnetic linear sensor is a robust, cost-effective sensor for linear position detection.

XKF3i is the proven sensor fusion algorithm used in the Xsens sensors, which fuses the information of the inertial and magnetic sensors and combines the advantages of different sensors to obtain accurate results for orientation estimation. In this way, the orientation quaternion value can be obtained by onboard calculation, the output of which can be used for further high-level calculation. The pitch/roll estimation accuracy is $1.0^{\circ }$, while the heading accuracy is $2.0^{\circ }$ for the 9-axis IMU [7]. Internal corrections for calibration errors are applied for the IMU sensors [8, 9].

The sensing system for the Hamlyn CRM is viewed in Fig. 4. One IMU (IMU A) is mounted on the handheld end-effector controller to sense its orientation, while another one (IMU B) is placed on the linear linkage to track its orientation. The magnetic linear sensor together with a multi-pole magnetic strip is fixed on the linear linkage to measure the relative linear motion of the prismatic joint. The end effector joins with the prismatic joint via a ball joint G. One Hall sensor (Hall Sensor A) is fixed on the gripper to sense the gripping motion together with a magnet at one of the jaws.

As for the end-effector orientation, the Euler angle can be calculated based on the IMU A, where $\varphi$, $\theta$ and $\gamma$ represent yaw, pitch and roll angle of the IMU A on its body frame B1, respectively. In order to help eliminate the ill-effects of drift in position estimation, additional sensors are utilized while an automatic reinitialization mechanism is introduced.

As for position estimation, the position value can be obtained by the double integral of the acceleration data, provided that the magnitude of the acceleration value is higher than that of the noises. In short-term position estimation, the positional estimation errors are relatively small, since the inertial sensor has high update frequency. The noises will propagate through the integration process, which leads to accumulative errors and the degradation during long-term position estimation process. Therefore, it is necessary to add additional sensor to combine the information from multiple sensors to realize accurate and stable position estimation.

For the Hamlyn CRM, the real-time position vector of the master manipulator end-effector $P_\mathrm{e}$ is defined in the master manipulator base frame M. $P_\mathrm{e}$ can be obtained through the forward kinematic based on the sensor information obtained from IMU B and the magnetic linear sensor. $\alpha$ and $\beta$ are the two serial rotation angles for the universal joint obtained by the IMU B on the linear shaft in its body frame B2. *L* is the joint length of the prismatic joint measured by the linear magnetic sensor. In this way, the information of $\theta _{1} = {\alpha }$, $\theta _{2} = {\beta }$ and $d = L$ can be obtained. Combining the 3D orientation and 3D position estimation results, the motion tracking of the 6D pose of the end effector can be realized.

**Fig. 5:**
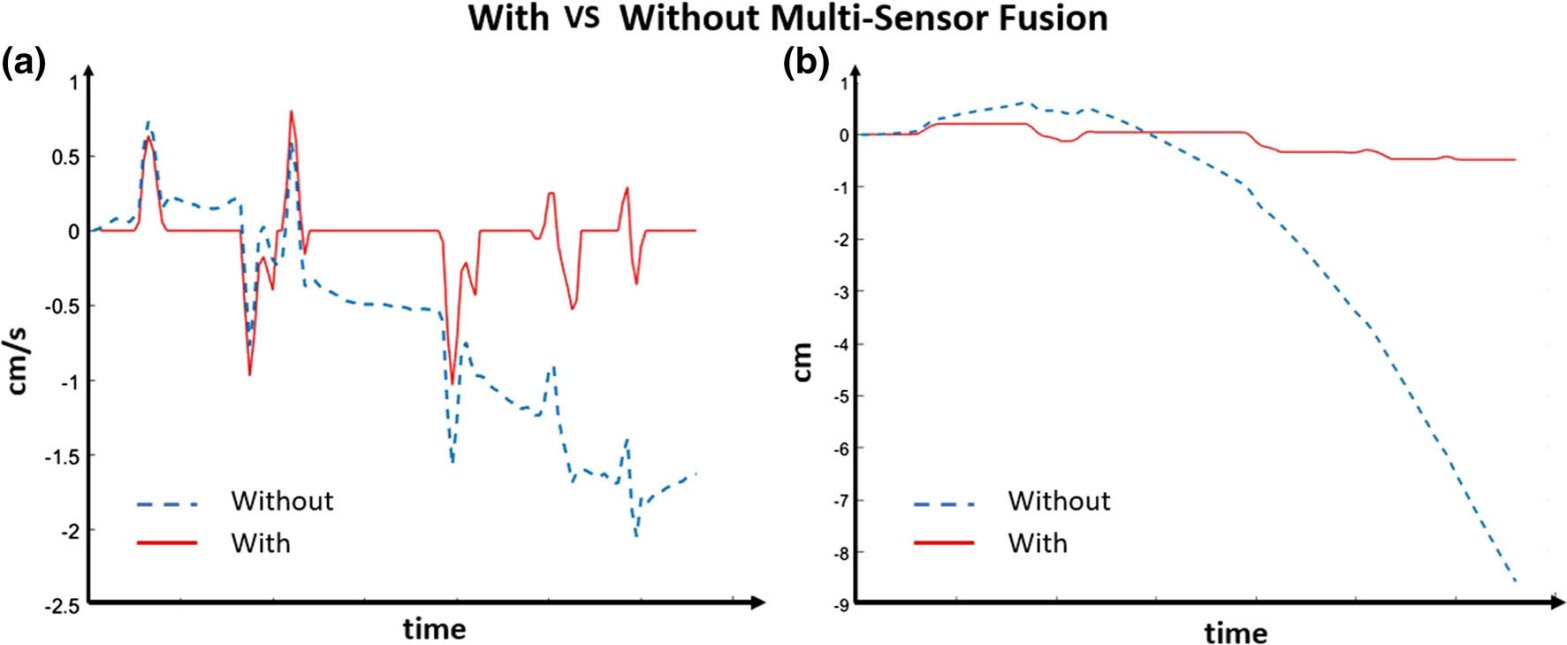
The comparisons between with and without pose estimation with multiple sensors. Comparisons between **a** velocity estimation; **b** position estimation

**Fig. 6:**
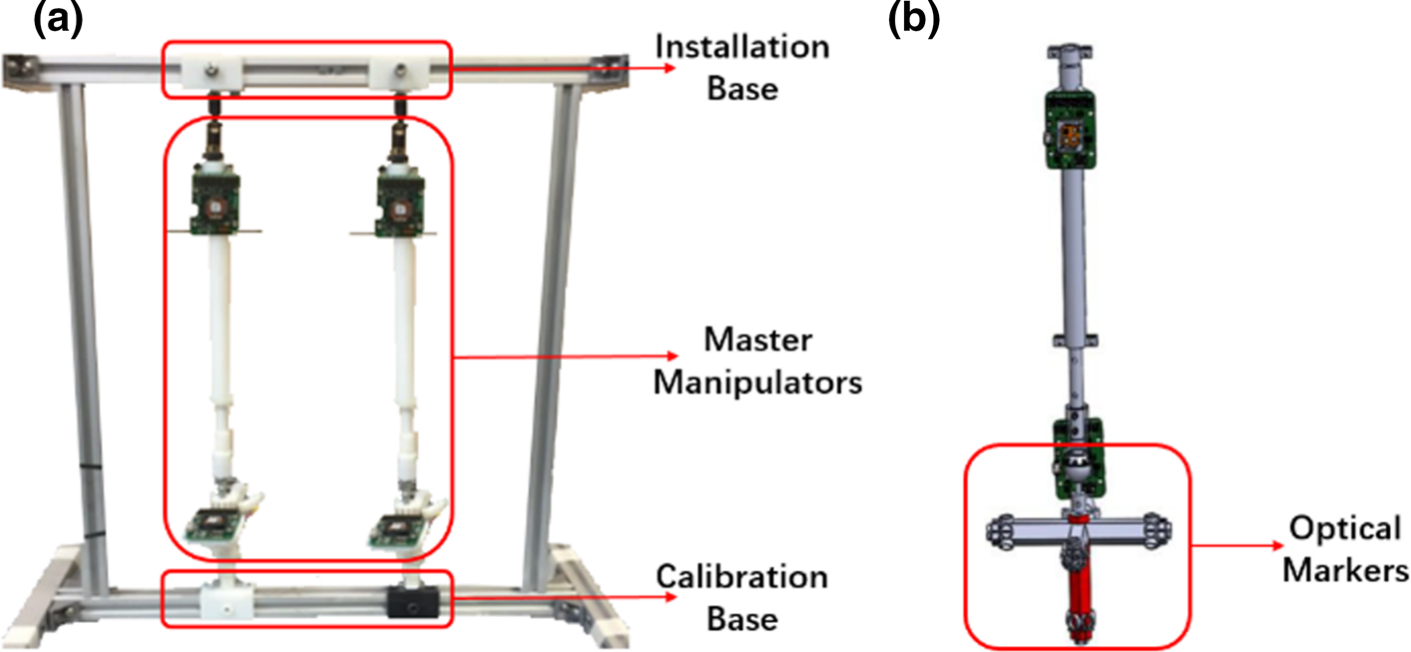
Prototype and CAD model for motion tracking experiments. **a** The physical prototype of the Hamlyn CRM; **b** the equivalent model for ground truth data acquisition using the optical tracking system

Comparisons are made between with and without using multiple sensors for pose estimation to verify the effectiveness of the overall sensing system. A series of IMU data were collected by controlling the end effector to conduct repetitive translational motion. The velocity and position of the end effectors were estimated by using a single IMU or using multiple sensors. Results are demonstrated in Fig. 5. It can be concluded that pose estimation with multiple sensors, the drift problem of using single IMU measurement by integrating the acceleration data can be addressed.

In order to further improve the motion tracking accuracy, an automatic reinitialization mechanism is utilized. The gripping angle $\sigma$ of the gripper is proportional to the output value of the Hall Sensor A. Another Hall sensor (Hall Sensor B) is installed at the bottom of the universal joint. The Hall Sensor B is used to detect whether the linear shaft is approached the joint limit or not. h is the distance between the magnet and the Hall Sensor B, $\epsilon$ is a threshold value. Once $h<\epsilon$ is detected, it can be confirmed that the linear shaft reaches joint limitation and the calibration mechanism is conducted automatically. During this process, the estimated link length is reset to the initial value (the minimal link length).

### Experiment results and analysis

The physical prototype of the Hamlyn CRM is shown in Fig. 6a. NDI optical tracking system (Northern Digital Inc., Canada) was used to obtain ground truth data and compare with the motion tracking results provided by the master manipulators. As shown in Fig. 6b, an equivalent model is built for the optical tracking experiment. The optical markers were mounted on the end effector.

Two types of tests were conducted, including the motion tracking accuracy comparisons during dexterous operation and non-dexterous operation. Dexterous operation means that the movements of the end effector change significantly during the test, while non-dexterous operation indicates that the movements of the operator change slightly and slowly during the experimental trials. The time length for data collection of each trial was set to be 3 s.

Suppose that ${{\varvec{Pg}}_{{{\varvec{1:T}}}}} {=} [Xg_{1:T},Yg_{1:T},Zg_{1:T}]$ and ${{\varvec{Og}}_{{{\varvec{1:T}}}}} = [{\Phi }g_{1:T},{\Theta }g_{1:T},{\Gamma }g_{1:T}]$ represent the position profile and the orientation profile generated by the ground truth data, respectively, with the total time length of *T* with sampling frequency of $f=30hz$. While ${{\varvec{Pr}}_{{\varvec{1:T}}}} = [Xr_{1:T},Yr_{1:T},Zr_{1:T}]$ and ${{\varvec{Or}}_{{\varvec{1:T}}}} = [{\Phi }r_{1:T},{\Theta }r_{1:T},{\Gamma }r_{1:T}]$ represent the position profile and the orientation profile obtained by the sensing system of the Hamlyn CRM. In this case, $T = 3$ s.

**Table 1:** Results for motion tracking

	Non-dexterous operation	Dexterous operation
PPD (mm)	$0.49 \pm 0.23$	$0.64 \pm 0.34$
OPD (rad)	$0.03 \pm 0.01$	$0.09 \pm 0.04$

**Fig. 7:**
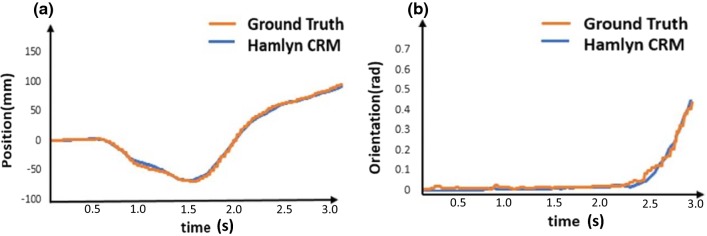
Motion tracking results comparison. Motion tracking results comparison between the sensing system of the Hamlyn CRM and the optical tracking system in terms of **a** position estimation; **b** orientation estimation

The Positional Precision Deviation (PPD) and the Orientational Precision Deviation (OPD) of the Hamlyn CRM estimated during each trial can be calculated as follows.

3 $$\begin{aligned} \hbox {PPD}&= \frac{ \sqrt{\sum _{t=0}^{t=T}{(Xg(t)-Xr(t))^{2}}} + \sqrt{\sum _{t=0}^{t=T}{(Yg(t)-Yr(t))^{2}}} + \sqrt{\sum _{t=0}^{t=T}{(Zg(t)-Zr(t))^{2}}}}{3Tf}\end{aligned}$$

4 $$\begin{aligned} \hbox {OPD}&= \frac{\sqrt{\sum _{t=0}^{t=T}{({\Phi }g(t)-{\Phi }r(t))^{2}}} + \sqrt{\sum _{t=0}^{t=T}{({\Theta }g(t)-{\Theta }r(t))^{2}}} + \sqrt{\sum _{t=0}^{t=T}{({\Gamma }g(t)-{\Gamma }r(t))^{2}}}}{3Tf} \end{aligned}$$

Five trials of motion tracking data were collected. The results of PPD and OPD obtained during different operation types are viewed in Table. 1.

Figure 7 shows one of the examples of the comparison of motion tracking results between the Hamlyn CRM sensing system and the optical tracking system which provides ground truth data. As shown in the figure, there are no significant differences between the motion tracking results obtained by the Hamlyn CRM sensing system and the ground truth data. The trajectory of the master manipulator is smooth without a sudden jump, which indicates that the consistent smooth control can be realized.

The tracking accuracy is sufficient for human-in-the-loop teleoperation, where motion tracking error, including slow drifts, can be corrected by the human’s visual feedback loop.

## Mapping strategy

### System integration

In order to verify the effectiveness of the master manipulator proposed in this paper, user studies were conducted on the dVRK [10]. The patient-side manipulators (PSMs) and the endoscope were utilized as the slave robot system.

**Fig. 8:**
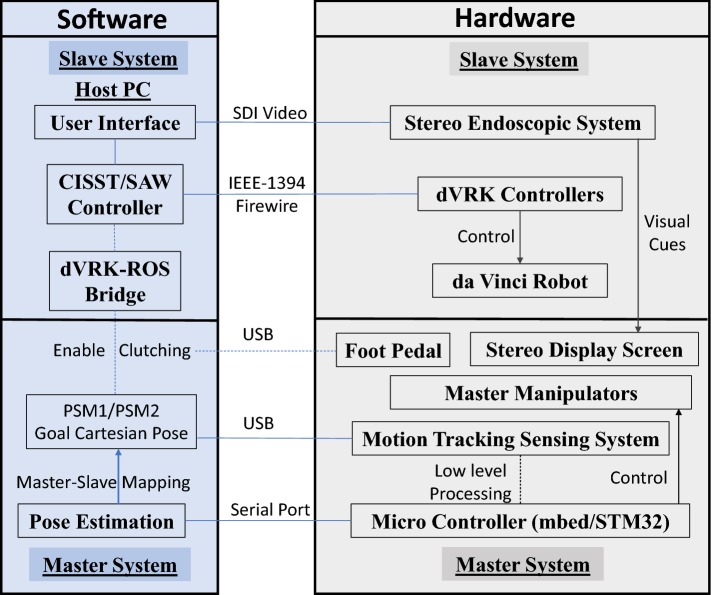
The framework for system integration of the hardware and software of the Slave robot system and master system

The system integration of the master manipulators and the dVRK slave robot, including the hardware system and software system, is illustrated in Fig. 8. High-level command generation is accomplished by a host PC, while low-level sensor data processing is fulfilled by micro-controllers (Arm Mbed OS). A foot pedal (Philips LFH2310) is used to generate the “engage” and “clutch” commands during teleoperation. All the sensor information is published as ROS topics and control the end-effector pose of PSM1 and PSM2 in the Cartesian space via the dVRK-ROS Bridge [10].

**Fig. 9:**
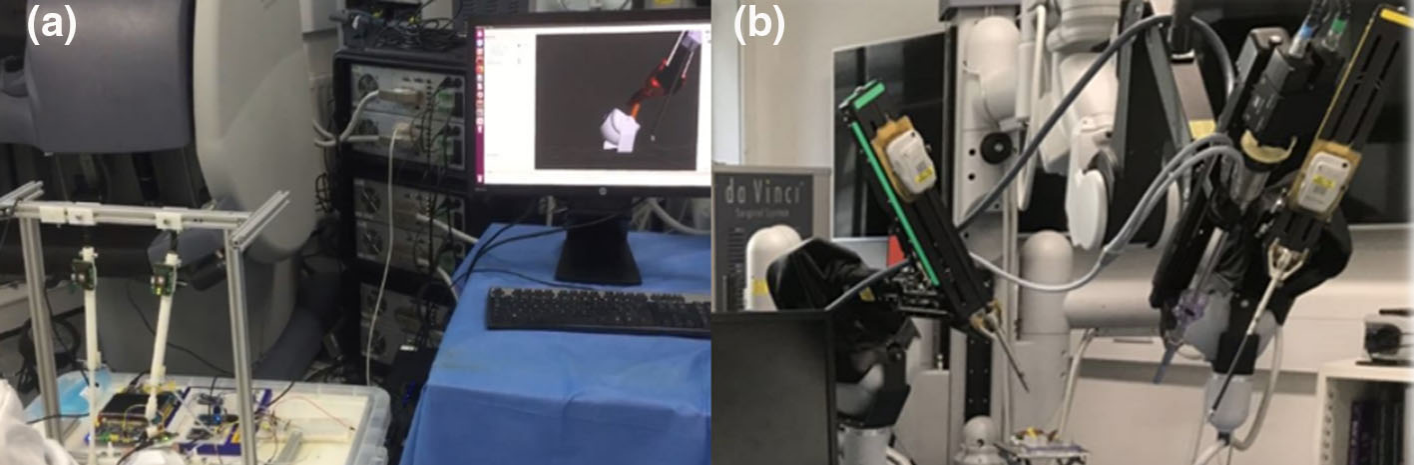
System integration of the Hamlyn CRM and the dVRK. **a** A user held one of the master manipulators at a typical pose and verify the control through simulation. **b** The da Vinci Robot is utilized as the slave robot during user studies

For the control system, a micro-controller (Arm Mbed OS) is used to acquire the sensor’s data and fulfill low-level sensor data processing. A foot pedal (Philips LFH2310) is utilized to provide the “engage” and “clutch” information during the remote control, similar to the control console of dVRK. Developed from the CISST-SAW, the dVRK robot’s end-effector Cartesian pose can be controlled via the dVRK-ROS Bridge [10].

The system integration of the Hamlyn CRM and the dVRK is viewed in Fig. 9. The operator can control a virtual da Vinci Robot for the basic evaluation of the motion tracking and the mapping strategy, while detailed user studies were conducted on the physical da Vinci Robot.

**Fig. 10:**
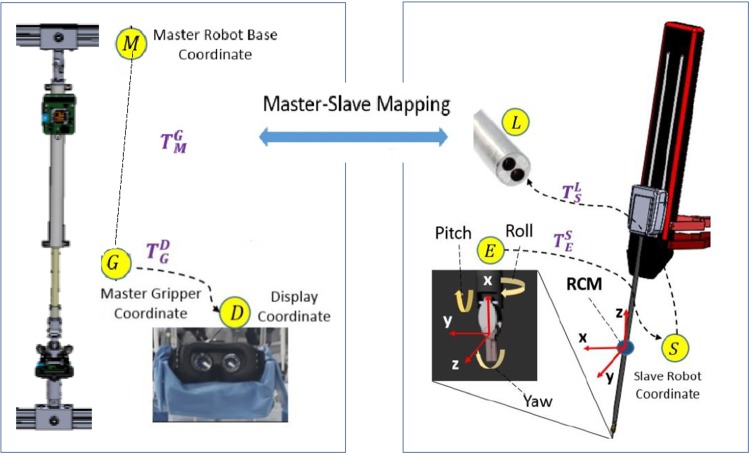
A description of coordinate system and the transform relationship among different frames of the master–slave system

**Fig. 11:**
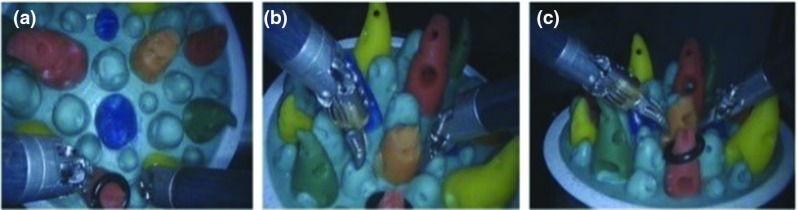
User studies for the hand–eye coordination. **a**$\partial \ =10^{\circ }\pm 5^{\circ }$; **b**$\partial \ =40^{\circ } \pm 5^{\circ }$; **c**$\partial \ =70^{\circ } \pm 5^{\circ }$

The overview of the master control console and the slave robot system is shown in Fig. 10. Frame D indicates the stereo vision coordinate of the display system in the master control console, which displays the stereo images obtained from the endoscope (frame L) and provides visual feedback to the operation during remote control. Frame G and frame E represent the master gripper coordinate and the slave robot end-effector coordinate, respectively. $T_\mathrm{M}^\mathrm{G}$ is the homogeneous transform matrix of the master gripper coordinate to the master manipulator base frame M, which is formed by translation matrix $P_\mathrm{M}^\mathrm{G}$ and rotation matrix $R_\mathrm{M}^\mathrm{G}$. It can be obtained through forward kinematics based on the DH table of the Hamlyn CRM.

The main target of the master–slave mapping determination is to find out the $T_\mathrm{S}^\mathrm{E}$ for slave robot control, which is the homogeneous transform matrix of the slave robot end effector to frame S (the remote center of motion of the slave robot). It is comprised of the translation matrix $P_\mathrm{S}^\mathrm{E}$ and the rotation matrix $R_\mathrm{S}^\mathrm{E}$. As for the world coordinate, the *z*-axis is coincided with the gravity direction, while the *x*-axis is pointed toward the operator and *y*-axis is defined by the right-handed coordinate. $T_\mathrm{G}^\mathrm{D}$ is the homogeneous transform matrix of the display coordinate in the master control console and the gripper coordinate. $\delta$ and $\partial$ are defined as the angle between the *z*-axis of the world coordinate and the central line of the visual display and the endoscope, respectively.

### User studies design

#### Participants

Seven subjects (two females and five males) were invited to participate in all the user studies. All the participants are right-handed. Each of the subjects has more than 5 min practice section to get familiar with the dVRK system before attending the formal user studies.

#### Key parameters selection

Hand–eye coordination is the ability to conduct activities that require the simultaneous use of the operators’ hands and eyes, which is significant for teleoperation. The operators need to generate commands based on the visual feedback of the remote scene through the vision system. In order to verify the importance of hand–eye coordination, user studies based on a ring transfer task were conducted to find out a suitable value of $\partial$ to form the $T_\mathrm{E}^\mathrm{L}$. $\partial$ is the rotation angle that generates $T_\mathrm{E}^\mathrm{L}$, which is the transformation matrix from the coordinate of the endoscope (frame L) to the coordinate of the slave robot end effector (frame E). Based on the consideration of ergonomics and the intuitiveness of operation [11], $T_\mathrm{G}^\mathrm{D}$ is generated by the rotation angle of $\delta =40^{\circ }\,{\pm }\,10^{\circ }$.

**Fig. 12:**
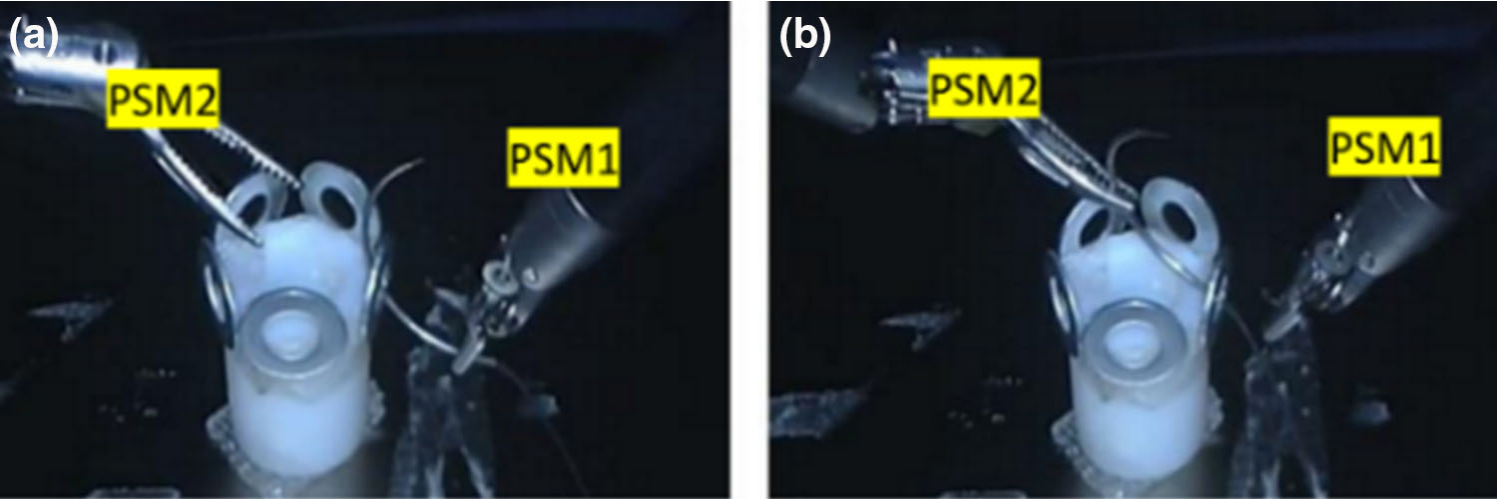
The experimental platform and scenes for user studies based on the needle passing task. **a** Insert needle. **b** Pull out needle

The task requires to transfer a ring through a pre-defined protocol. Figure 11 shows different views of the endoscope collected during the user studies. The kinematics data of the master and slave robots were both recorded for further performance evaluation. After determining the reasonable value of $\partial$, the second user study for the motion scaling ratio selection was conducted.

**Fig. 13:**
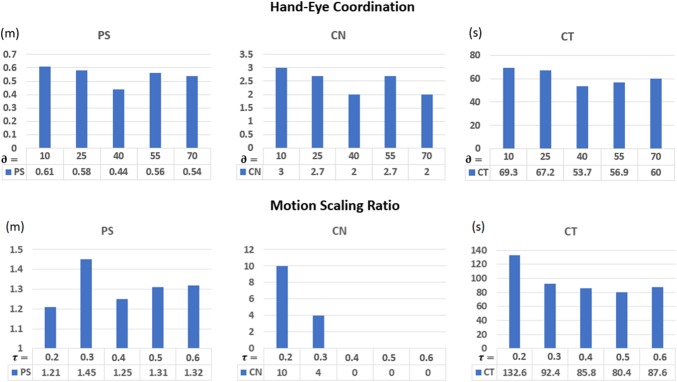
User study results for the key parameters selection

In robotic surgery, motion scaling represents the conversion of the surgeon’s large hand movements into smaller movements of the surgical instrument in the operative field. This means that the position mapping during teleoperation requires to map the position change of the master to the slave by a scaling factor $\tau$, through which precise operation can be achieved. Here, we denote ${{\varvec{PS(t)}}}$ and ${{\varvec{PM(t)}}}$ as the slave robot and the master robot’s end-effector position at time step *t*, respectively, $\tau$ as the motion scaling factor, ${\Delta }t$ as the time interval for control, *t* represents the time step.

5 $$\begin{aligned} {{{\varvec{PS(t)}}} = {\tau }({{\varvec{PM(t)}}} - {{\varvec{PM(t-1)}}}) + {{\varvec{PS(t-1)}}}} \end{aligned}$$

#### Master–slave mapping mode

Considering that the master and the slave robot are heterogeneous, instead of using joint-to-joint mapping, the end-effector mapping is utilized to deal with the mismatch between the workspace of the master and the slave robot. In pursuit of higher precision, P mode is used for the position mapping [6]. As for orientation mapping, two types of master–slave mapping mode are explored in this paper. The absolute mode for orientation mapping indicates that the orientation of the slave robot’s end effectors is the same as the master manipulators’ end effectors, while the relative mode indicates that the increment value of the orientation of the slave robot’s end effectors is the same as the increment value of the master manipulators’ end effectors.

The absolute mapping can ensure the sense of intuitiveness, while the relative mapping mode can enable the user to adjust to a suitable pose easily, for the sake of ergonomics. Since they have relative advantages, user studies for further determination are necessary.

To compare the two orientation mapping strategies, the third user study was based on a needle passing task, which has higher requirements on the orientation control. This task requires the subjects to pass a suture needle through two holes, including two repetitive bimanual operations, i.e., bringing the needle toward the holes, using one arm to insert the needle (see Fig. 12a) and another arm pull it out (see Fig.  12b). The end of the task is determined by the needle passing the hole completely.

### Experiment results and analysis

For the quantitative analysis, three evaluation metrics are employed to evaluate the performance of the subjects during user studies. These include the path length of the slave robot’s end-effector trajectory (PS), the number of clutching (CN) and the completion time (CT) during a single trial.

For the hand–eye coordination parameter selection, $\partial$ ranging from $10^{\circ } \pm 5^{\circ }$ to $70^{\circ } \pm 5^{\circ }$ were studied. Based on the user study results summarized in Fig. 13, $\partial \ =40^{\circ }\,{\pm }\,5^{\circ }$ is the best angle range for placing the endoscope with respect to the z-axis. For the motion scaling ratio selection, $\tau$ ranging from 0.2 to 0.6 were studied in this paper. Results are found in Fig. 13. $\tau =0.4$ is selected, since it contributed to the best performance in terms of the PS and CN, while the CT is comparable to $\tau =0.5$.

The results of the comparison of the orientation mapping are viewed in Table 2. Based on the results, the absolute mapping is better than the relative mapping, since it contributed the best results among all the three evaluation metrics. The feedback from subjects also indicated that absolute mapping is better, which is more intuitive and demands fewer efforts for teleoperation.

**Table 2:** Results for the orientation mapping mode selection

	Absolute mode	Relative mode
PS (m)	$0.97\,\pm \,0.29$	$1.14\,\pm \,0.24$
CN	$3\,\pm \,2$	$4\,\pm \,2$
CT (s)	$87.4\,\pm \,15.6$	$90.6\,\pm \,18.6$

To summarize, the position relative mapping mode and the orientation absolute mapping mode are combined to form the mapping strategy for surgical robot remote control via the compact master manipulator. $T_\mathrm{S}^\mathrm{E}$ at time step *t* can be defined by ((6)), where $\partial =40^{\circ }\,{\pm }\,5^{\circ }$ and $\tau =0.4$ are the desired parameters for hand–eye coordination and motion scaling, respectively. $T_\mathrm{S}^\mathrm{E}$ can be obtained based on the input value of $T_\mathrm{M}^\mathrm{G}$ generated by the master manipulator.

${{\varvec{T}}}_\mathrm{S}^\mathrm{E}$ is the final result that used to control a slave robot. Here, we define ${{\varvec{R}}}_\mathrm{M}^\mathrm{G}$ and ${{\varvec{P}}}_\mathrm{M}^\mathrm{G}$ as the rotation matrix and the translation vector that form the homogeneous transform matrix ${{\varvec{T}}}_\mathrm{M}^\mathrm{G}$. ${{\varvec{T}}}_\mathrm{S}^\mathrm{E}$ at time step *t* can be obtained as follows.

6 $$\begin{aligned} {{\varvec{T}}}_\mathrm{S}^\mathrm{E}(t)= \begin{bmatrix} {{\varvec{R}}}_\mathrm{M}^\mathrm{G}(t) &{} {{\varvec{P}}}_\mathrm{S}^\mathrm{E}(t-1) + \tau ( {{\varvec{P}}}_\mathrm{M}^\mathrm{G}(t) - {{\varvec{P}}}_\mathrm{M}^\mathrm{G}(t-1))\\ {{\varvec{0}}} &{} 1 \end{bmatrix}\nonumber \\ \end{aligned}$$

Thus far, the mechanical design, the motion tracking system for remote control and the master–slave mapping strategy have been illustrated.

To further verify the overall properties of the Hamlyn CRM, comparisons were made with another commercial compact robotic master (Phantom Omni), while the comparisons with the non-compact dVRK Master Tool Manipulators (MTMs) were used as the reference. The Hamlyn CRM and the Phantom Omni were integrated into the dVRK control console for user studies. Therefore, the stereo vision system of the dVRK control console was used to provide visual feedback. In this way, the user study results were only influenced by the design of the master manipulators. Comparisons are made in terms of PS, CT, CN and AV (average velocity) based on a ring transfer task.

**Fig. 14:**
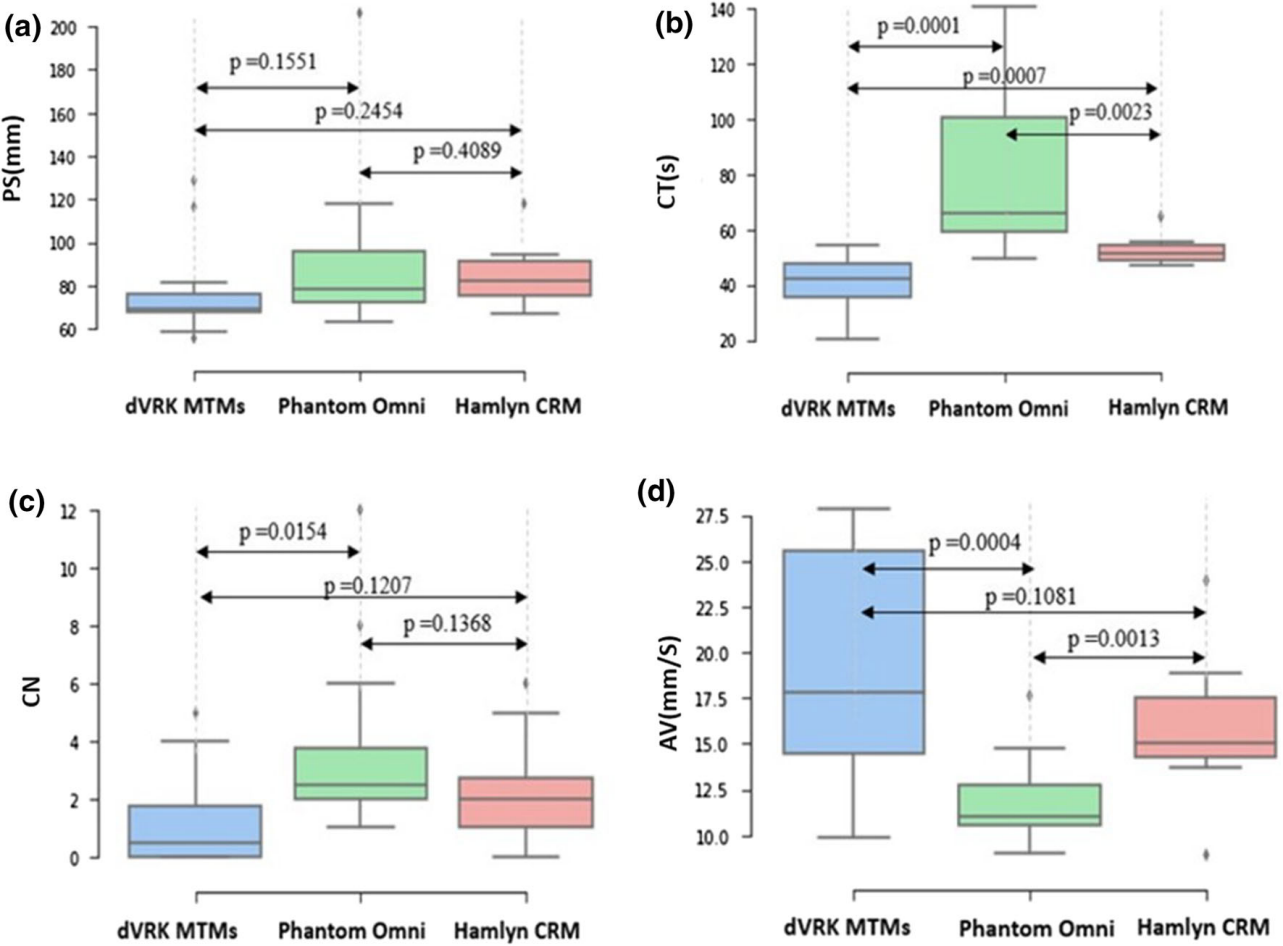
Statistical Analysis of Experiments (p:*p* value). **a** Comparisons in terms of slave robot path length (PS); **b** task completion time (CT); **c** clutching number (CN); **d** average velocity (AV)

Among all the performance metrics, the Hamlyn CRM has a relatively short total trajectory of the slave robot during operation, and less clutching number and task completion time, compared to the Phantom Omni, while the average speed is higher. This indicates that the performance of the Hamlyn CRM is better than the Phantom Omni among all evaluation metrics. The results for each master manipulator are plotted in a box plot form, as shown in Fig. 14.

For the results derived, the t-test is applied. The *p* values are calculated for different metrics between two different master manipulators, as shown in Fig. 14. It can be concluded that PS does not have a significant contribution (*p* value $> 0.05$) to differentiate the performance of different master manipulators. There are significant differences between the Hamlyn CRM and the Phantom Omni in terms of CT ($p\, \hbox {value} = 0.0023\,<\,0.005$) and AV ($p\, \hbox {value} = 0.0013\,<\,0.005$). With regard to CN, the difference is not significant ($p\, \hbox {value} > 0.05$); however, significant difference between the Phantom Omni and the dVRK MTMs is observed ($p\, \hbox {value} = 0.0154 < 0.05$), while the difference between the Hamlyn CRM and dVRK MTMs can be overlooked ($p\, \hbox {value} = 0.1207 > 0.05$). The difference of the average velocity between the Hamlyn CRM and the dVRK MTMs can be overlooked ($p\, \hbox {value} = 0.1081>0.05$).

User studies indicated that the Hamlyn CRM can be utilized for surgical procedures and has a high potential for surgical robot remote control.

## Discussions

Precise motion tracking is important for the master manipulator to realize reliable remote control. The experiment has verified the reasonable tracking accuracy, but the advantages of the proposed design, compared to completely freehand systems, need to be considered for future development. The motion tracking accuracy during dexterous operation is worse than the estimated accuracy during non-dexterous operation. Therefore, machine learning-based method will be explored for pattern recognition with adaptive Kalman filter to enable the end-effector pose estimation to be more stable and precise during dexterous operation.

As for the exploration of master–slave mapping strategy, the optimal parameters may be slightly changed based on the specific applications as well as the expertise levels of the subjects. For example, a fine dissection task may benefit from a high scaling ratio, while a gross manipulation of bowel may benefit from a lower scaling ratio. Since the major targeted applications are for laparoscopic surgery, the surgical operations mostly include procedures like needle passing, object transfer and suturing. The selected parameters can remain within a general range. This paper focuses on the new design of a master manipulator, while the key parameters for master–slave mapping and the mapping strategy are explored. Adaptive shared control can be implemented through an adaptive motion scaling framework to further enhance the usability and operation efficiency through the master manipulator [12].

The Hamlyn CRM has a comparative performance to the dVRK MTMs and outperforms Phantom Omni according to the user study results. The task completion time reflects the overall difficulty of the task. Shorter time indicates the master controller is simpler for users. The clutching number reflects the necessity to disconnect the master manipulators from the slave robot for position adjustment. Higher clutching number reveals a significant mismatch between the master and the slave robot’s workspace, which leads to the reduction in control efficiency during teleoperation. As for the path length of the slave robot end-effector trajectory, a higher value indicates that operators required more efforts to control the slave robot. Smaller total path length is preferred, since the reduced straightness of the navigation path for slave robot control can be observed.

The task completion time for the dVRK MTMs is the shortest, the clutching frequency is the lowest, while the average velocity for task fulfillment is the highest, which may due to the fact that some of the users are familiar with dVRK and have more experience in using it. In addition, the dVRK MTMs are much more reliable, compared to the 3D printing version of the Hamlyn CRM. Since the dVRK MTMs is non-compact design, the manipulators have significant larger dimensions to generate larger workspace. Unsurprisingly, the clutching number can be reduced. These factors may be potential influence factors for the user studies, but the results are still valuable to evaluate the overall performance of the Hamlyn CRM and demonstrates its potential applications as a compact master manipulator for robotic surgery.

## Conclusions

In this paper, we have developed a compact master manipulator, with detailed illustration of the structure design, the sensing system, the motion tracking framework and the selection of the master–slave mapping strategy. Optical tracking was employed to obtain the ground truth data for comparison between the motion tracking results obtained from the Hamlyn CRM. Results indicate that the trajectory profile is smooth, and the consistent control can be implemented. The suitable motion scaling ratio value, hand–eye coordination parameter and mapping paradigm are determined through user studies based on a ring transfer task and a needle passing task in this paper.

The Hamlyn CRM has the similar performance of the dVRK MTMs, while having evident strengths compared to the Phantom Omni. The feedback from the user study also proved that the Hamlyn CRM has high manipulability for intuitive control of the surgical robot and is portable thanks to the compact structure. It outperforms the Phantom Omni in terms of the completion time, average velocity and clutching number and has a comparative performance to that of the dVRK MTMs, thus demonstrating the potential clinical value of the device.

The Hamlyn CRM has high potential to be applied for surgical training. In addition to using the Hamlyn CRM for ring/peg transfer task, needle passing task, it can be used for other more advanced surgical training such as knot tying and suturing. Since it is a compact master manipulator and has stable motion tracking, it may have high potential to be utilized in a tiny clinical center for conducting surgical procedures.

Future work will include evaluating the effectiveness of the master manipulator when compared with other mature commercial products. Ergonomics design consideration will also be discussed in the future.
